# Screening of PDSS1 as a Potential Biomarker for Hepatocellular Carcinoma Based on a Copper-Related Prognostic Signature through Bulk and Single-cell RNA-sequencing Analysis

**DOI:** 10.7150/jca.96867

**Published:** 2024-07-22

**Authors:** Kainan Lin, Jingwei Cai, Siyuan Pan, Xuefei Yu, Yiwu Qiu, Zhangguo Ying, He Feng, Lizhuo Zhang, Yanyang Liu, Huize Shen, Yangjian Hong, Qinglin Li, Renan Jin

**Affiliations:** 1 Zhejiang University School of Medicine, Zhejiang University, Hangzhou 310058, Zhejiang Province, China.; 2 Department of General Surgery, Sir Run-Run Shaw Hospital, Zhejiang University, Hangzhou 310016, Zhejiang Province, China.; 3 Zhejiang Cancer Hospital, Key Laboratory of Head & Neck Cancer Translational Research of Zhejiang, Hangzhou 310022, Zhejiang Province, China.; 4 School of Basic Medical Science, Zhejiang Chinese Medical University, Hangzhou 310053, China.

**Keywords:** liver hepatocellular carcinoma, cuproptosis -related prognostic signature, PDSS1, tumor-associated macrophages, tumor-related transcription factors, high metabolic microenvironment

## Abstract

**Background:** Currently, there is few literature comprehensively analyzing landscape of cuproptosis-related genes (CRGs) in liver hepatocellular carcinoma (LIHC) with multiple omics approaches.

**Aims*:*** Using comprehensive analysis, we aim to find out how CRGs works on LIHC.

**Method:** With data from The Cancer Genome Atlas (TCGA) database, we constructed a prognostic prediction model for CGRs using LASSO regression analysis and performed immune infiltration analysis using the same dataset. To validate findings, we utilized RNA expression data from the International Cancer Genome Consortium (ICGC). Furthermore, we analyzed the enrichment and features of CRGs in epithelial cells using single-cell RNA sequencing (scRNA-seq) data. To validate the reliability of findings, we performed several experiments including RT-PCR, cloning formation assay, scratch assay, and Transwell assay.

**Result:** We have constructed a high-precision risk scoring model composed of CRGs for predicting prognosis in TCGA-LIHC. Reliability of the risk prognosis model was confirmed through Kaplan-Meier curve analysis, time-dependent ROC analysis, and multivariate regression analysis. Furthermore, we found knocking down PDSS1 increased sensitivity of LIHC cells to copper ions, and both proliferation and migration abilities were significantly reduced. Finally, we comprehensively characterized the features of CRGs in LIHC through scRNA-seq.

**Conclusion:** In this study, we introduce PDSS1 as a novel CRG in HCC. Utilizing scRNA-seq, we provide a comprehensive landscape of cuproptosis across various cell subtypes within the HCC tumor microenvironment. Furthermore, we detailed the characteristics of high PDSS1-expressing tumor cells, including their distinctive transcription factors, metabolic profiles, and interactions with different subtypes within the tumor microenvironment. This work not only elucidated the role of PDSS1 in HCC but also enhanced our understanding of cuproptosis dynamics during tumor progression.

## Introduction

LIHC is one of the most common and lethal cancer worldwide, with a 5-year survival rate of approximately 18, about 830,000 deaths each year, representing it one of the leading causes of cancer-related deaths globally 1. Despite significant advancements in diagnostic techniques and treatment strategies for LIHC in recent years, improvement in the prognosis of LIHC patients is still limited 2,3. LIHC is not only a conglomerate of malignant proliferating cells, but also a complex tumor microenvironment consisting of immune cells, stromal cells, endothelial cells, and cancer-associated fibroblasts, among others. This further contributes to the complexity of LIHC treatment and early tumor recurrence 2. Tumor associated macrophages (TAMs) are the main inflammatory cells in the tumor microenvironment. They can suppress anti-tumor immunity, promote tumor angiogenesis, and alter tumor metabolic status 3. T cells can differentiate into various subtypes and coordinate various immune responses, targeting and destroying tumor cells, or inhibiting anti-tumor immune responses through the secretion of cytokines 4. Therefore, there is an urgent need to identify novel effective biomarkers and integrate comprehensive analyses of single-cell transcriptomics and spatial transcriptomics for early detection and risk prediction, to improve patient prognosis.

Copper is an essential trace element in human body, serving as a cofactor for various metabolic enzymes and playing a crucial role in multiple biological processes. However, inappropriate concentrations of copper can be toxic to cells and associated with various diseases, including Wilson's disease, hepatolenticular degeneration, and the occurrence and development of tumors 5. In liver cells of patients with Wilson's disease, it has been found that autophagy-related genes are upregulated in response to intracellular copper overload, thereby preventing copper-induced cell apoptosis 6. Additionally, studies have found a significant relationship between copper ion levels in the blood and the occurrence of non-alcoholic fatty liver disease (NAFLD). Low copper concentration is an independent risk factor for the development of NAFLD in male patients with metabolic syndrome 7. Recently, a novel cell death mechanism has been discovered that is dependent on intracellular copper levels and differs from other known cell death types, named cuproptosis 8. This indicates that copper concentration may be an important mechanism affecting cancer progression. However, there is still little comprehensive analysis of these CRGs and their impact on the tumor microenvironment.

Currently, the rapid development of bioinformatics technology guarantees us more effective methods to comprehensively analyze molecular mechanisms and network relationships involved in LIHC prevention, diagnosis, and progression monitoring. With advancement of single-cell RNA-seq analysis, we can identify disease-relevant cell subtypes within tumor tissues in a high-resolution and unbiased manner. It allows us to comprehensively unravel tumor microenvironment in different states through a series of analyses 9. However, despite studies utilizing bioinformatics technology to investigate CRGs in LIHC, there is no research yet that comprehensively analyzes CRGs through integrating single-cell transcriptomics. This may provide a new avenue for further research on LIHC.

Therefore, we designed this study to employ a multi-omics approach to identify and elucidate function of CRGs in LIHC prognosis and remodeling of the tumor microenvironment. First, we constructed a LIHC prognosis prediction model based on the composition of CRGs. Additionally, we analyzed the TME of LIHC using expression profile data from TCGA and ICGC. Furthermore, we validated and expanded analysis of bulk sequencing data through experimental validation and single-cell transcriptomic analysis. These findings may contribute to a comprehensive understanding of the landscape of cuproptosis in LIHC, and potentially improve treatment outcomes and prognosis based on underlying molecular mechanisms.

## Materials and Methods

### Data sources

Data for LIHC were retrieved from two comprehensive genomic databases: TCGA and ICGC. Initially, the TCGA dataset comprised 374 samples, from which we systematically averaged data from repeated sequencing efforts. Furthermore, samples lacking complete clinical data were excluded, resulting in a final cohort of 365 samples for inclusion in our study. From the ICGC dataset, out of the initial 243 samples, those lacking mRNA expression matrices but containing only somatic mutation data were excluded. This filtering criterion led to the retention of 231 samples, which were subsequently used for validation purposes. **Table 1** listed the clinical data for all patients across the two cohorts. Transcriptome profiling from TCGA was used to quantify gene expression (RNAseq) and the results were harmonized to Fragments per Kilobase Million (FPKM). Training and validation were conducted using the TCGA dataset (N = 365) and ICGC dataset (N = 231) of LIHC patients, respectively. The TCGA cohort was utilized as the training set, and the ICGC cohort was employed as the external validation set. Our analysis of different gene expression was conducted using the Bioconductor limma package of R software (version 4.0.2).

### Consensus clustering analysis of copper metabolism-related genes

Two-hundred CRGs were retrieved from the Molecular Signatures Database, MSigDB (https://www.gsea-msigdb.org/gsea/msigdb). Detailed information on these gene sets can be found in **Table S1**. Consensus unsupervised clustering was performed to explore the potential molecular subtypes between the LIHC patients according to CRG expression using the R package “ConsensusClusterPlus.” The optimal number of clusters (K) was determined by commonly applied methodology including cumulative distribution function (CDF) and relative change in area under CDF curve.

### Establishment of CRG signature

The prognostic signature based on CRGs was performed in three steps: First, we used differential expression analysis to find the differential expression genes (DEGs) of copper metabolism. Second, we used Cox regression analysis to find the genes with significant differences in univariate analysis linked to LIHC OS. Then, a signature based on the LASSO Cox regression algorithm, employed to reduce the risk of over-fitting based on the “glmnet” R package, was developed in the training set using 64 copper metabolism related prognostic genes. To establish a prognostic CRG score, candidate genes were chosen using multivariate Cox analysis. The following formula was used to calculate the CRG score: CRG_score = Σ(Expi * Coefi) where the coefficient value and gene expression level were represented by Coefi and Expi, respectively. Depending on the median risk score, 374 patients in the training set were separated into high and low risk groups and carried out to Kaplan-Meier survival analysis. Accordingly, the validation set, also divided into low- and high-risk groups, was subjected to Kaplan-Meier survival analysis and receiver operating characteristic (ROC) curves.

### Development and validation of prognostic nomogram

Cox regression analysis was performed to screen clinical prognostic factors along with risk score status as the prognostic parameters to generate a nomogram model for predicting the probability of survival at 1, 3 and 5 years in LIHC patients and plotted the nomogram using the “rms” R packages. Calibration plots were employed to assess the discriminative ability of the nomogram. To estimate the accuracy of the actual observed rate with the predicted survival for 1-year, 3- and 5- year OS of the nomogram, the ROC curve and calibration curve fluctuating with time were also drawn.

### Immune profile analysis

We performed immune infiltration analysis using a deconvolution method, the CIBERSORT algorithm, to identify immune cell populations in both high-risk and low-risk cohorts from the TCGA and ICGC datasets. The standardized microarray profiles of the high-risk and low-risk groups were input into the CIBERSORT online analysis platform (https://cibersort.stanford.edu/) to obtain the corresponding immune cell composition profiles. Subsequently, statistical analysis was conducted to determine the differences in immune cell infiltration between the two risk groups, yielding reliable conclusions.

### Cell culture and transfections

The LIHC cell lines, namely HCC-LM3 and SK-Hep-1, were procured from the American Type Culture Collection and cultured in Dulbecco's Modified Eagle Medium supplemented with 10% (v/v) fetal bovine serum. The cells were maintained under optimal conditions at 37 °C in an environment with 5% CO2. Regular screenings confirmed the absence of mycoplasma contamination in all cell lines. For the introduction of siRNA and plasmids, Lipofectamine 3000 Reagents (Thermo Fisher Scientific, Waltham, MA) were utilized following the provided manufacturer's guidelines. The transfection procedure was performed in accordance with the recommended protocols.

### Colony formation assay

Cell clone formation was evaluated through a colony formation assay. In essence, a batch of tumor cells (1.0×10^3^ cells/60 mm culture dish) was seeded in triplicate and cultivated at 37 °C over a span of two weeks to facilitate the formation of distinct colonies. Subsequently, the cells were subjected to a PBS wash, followed by fixation with 4% paraformaldehyde for a duration of 15 minutes. A 30-minute staining procedure with a solution of crystal violet (comprising 1% paraformaldehyde, 0.5% crystal violet, and 20% methanol in PBS) ensued. The count of distinct colonies on each culture dish was tallied, serving as a quantifiable indicator of cell viability and survival capacity.

### Quantitative real-time PCR (qRT-PCR)

Total RNAs were meticulously extracted from tissues or cells using the TRIzol reagent. Subsequent to the extraction process, reverse transcription was diligently carried out utilizing the Takara RNA PCR kit, following the established guidelines furnished by the manufacturer. To quantitatively analyze the transcripts of specific target genes, real-time PCR was meticulously executed employing the Hieff UNICON® qPCR SYBR Green Master Mix. This amplification procedure was undertaken using the Roche LightCycler 480 QPCR apparatus within specialized 96-well QPCR plates, provided by Roche Diagnostics Corp. in Indianapolis, IN.

### Wound healing assay

To assess the migratory capacity of cells, we conducted the wound-healing assay. Cells that had undergone transient transfection were meticulously placed in both chambers (at a seeding density of 1 ~ 2 × 10^5^/well) within the wound healing assay chamber. This chamber was situated within a 6-well plate, and the experiment was carried out in triplicate. Once the cells adhered to the culture surface, the wound healing assay chamber was delicately removed. Subsequently, photographs of the spaces created between the two chambers were taken at intervals of 12 hours. This allowed us to closely monitor and document the progressive movement of the cells as they migrated to close the gaps.

### Migration assay

Cells transiently transfected were gently suspended in serum-free medium and subsequently introduced into the upper chamber of 24-well transwell inserts. These inserts featured 8 μm-pore size membranes and were obtained from Corning Inc. The cell seeding density ranged from 2-6 × 10^4^ cells per well. The assembled transwell chambers were incubated at 37 °C in an atmosphere containing 5% CO2, allowing for cell migration to take place over a span of 24 to 48 hours. Following the incubation period, the chambers were meticulously fixed using ice-cold 100% methanol for 20 minutes. Subsequently, staining was conducted using a solution of 0.1% Crystal Violet for an additional 20 minutes at room temperature. Each well was then imaged at a magnification of 20×. This experiment was performed three times to ensure consistency and reliability of the results. We have provided representative images to visually depict the outcomes of these assays.

### Dimension reduction and clustering analysis

We downloaded scRNA-seq data of LIHC from GSE149614 for further analysis. First, we performed quality control on the GSE149614 data based on the following criteria: nFeature_RNA > 200, percent.mt < 20, percent.HB < 5. Next, we applied the LogNormalize method to normalize the data. *FindVariableFeatures* function in R package “Seurat” v4.0.1 was applied to identify top 2000 most variable genes which was used to scale our data. Principal Component Analysis (PCA) was also performed based on the top 2000 most variable genes mentioned above. The *ElbowPlot* function in the R package “Seurat” was used to select the optimal number of principal components (PCs). Before identifying cell types, we utilized R package “Harmony” to eliminate batch effect. Based on selected number of PCs, we used the *FindNeighbors* function in the Seurat package to explore nearest neighbors and then performed cell clustering using the *FindClusters* function. Finally, we applied the uniform manifold approximation and projection (UMAP) algorithm for dimensionality reduction and visualization of cell subtypes. Cells were clustered into 10 types based on the gene markers.

### Gene and gene set expression analysis

We utilized the *AddModuleScore* function in Seurat package to score the CRGs and the gene set they comprised. Next, we visualized the enrichment levels of CRGs in different cells and tissues by *FeaturePlot* function and *VlnPlot* function.

### TF regulon analysis

The enrichment TFs and the regulatory network in cell subtypes was analyzed by R package “SCENIC” v1.3.1. We identified potential TFs by performing the *GENIE3* method based on the standard SCENIC analysis workflow. The regulon activity in each cel measured in AUC was determined by *AUCell* module of the R package “SCENIC”.

### Metabolic pathway identification

The R package “scMetabolism” v0.2.1 was employed to calculate the differences of metabolic state between epithelial cell types with different PDSS1 expressions. Based on the integrated metabolic gene sets from the Reactome database, we assessed the average gene expression of metabolic genes and median pathway scores for the two types.

### Cell-Cell interaction analysis

In order to investigate the potential interactions between epithelial cells with different PDSS1 expressions and tumor microenvironment cells, the R package “CellChat” v1.5.0 was utilized for the analysis of intercellular communication. Based on annotated cell gene expression profiling data, the abundance level of the interactions between the two cell types was inferred by analyzing the expression of ligands and receptors between cells. The *netVisual_circle* function was used to visualize the differences in network communication quantity and strength, while the *netVisual_bubble* function was utilized to display the intensity of communication between ligands and receptors.

### Pseudotime and trajectory analysis

We performed the pseudotime and trajectory analysis among epithelial cells by using R package “Monocle2” v2.16.0 with default settings. By applying *reduceDimension* and *orderCells* functions in Monocle2 package, we pseudo-temporally ordered cells. Next, the expression changes of CRGs along the trajectory of pseudotime were visualized using the *plot_genes_in_pseudotime* function. The cell density plot along the time axis was visualized using ggplot2 package.

### Pathway enrichment analysis

For bulk RNA-seq analysis, we employed R package “limma” v3.54.0 to identify the DEGs between high-risk and low-risk groups. Then, for the identified DEGs, we used the R package “clusterProfiler” v4.9.0.002 to perform gene ontology (GO) and Kyoto Encyclopedia of Genes and Genomes (KEGG) analysis. For scRNA-seq analysis, we identified globally DEGs of cell subpopulations using the* FindAllMarker* function in the Seurat package, based on the filtered gene expression matrix obtained from Seurat. To investigate the functional and mechanistic differences of epithelial cells with different PDSS1 expressions, we performed subsequent analysis using the R package “irGSEA” v2.1.5 on the Hallmark gene set from MSigDB (http://www.gsea-msigdb.org/gsea/index.jsp). This analysis was conducted to determine the enrichment of biological pathways in the clusters of the cells.

## Results

### Identification of copper metabolism-related prognostic genes in LIHC patients

**Figure 1:**
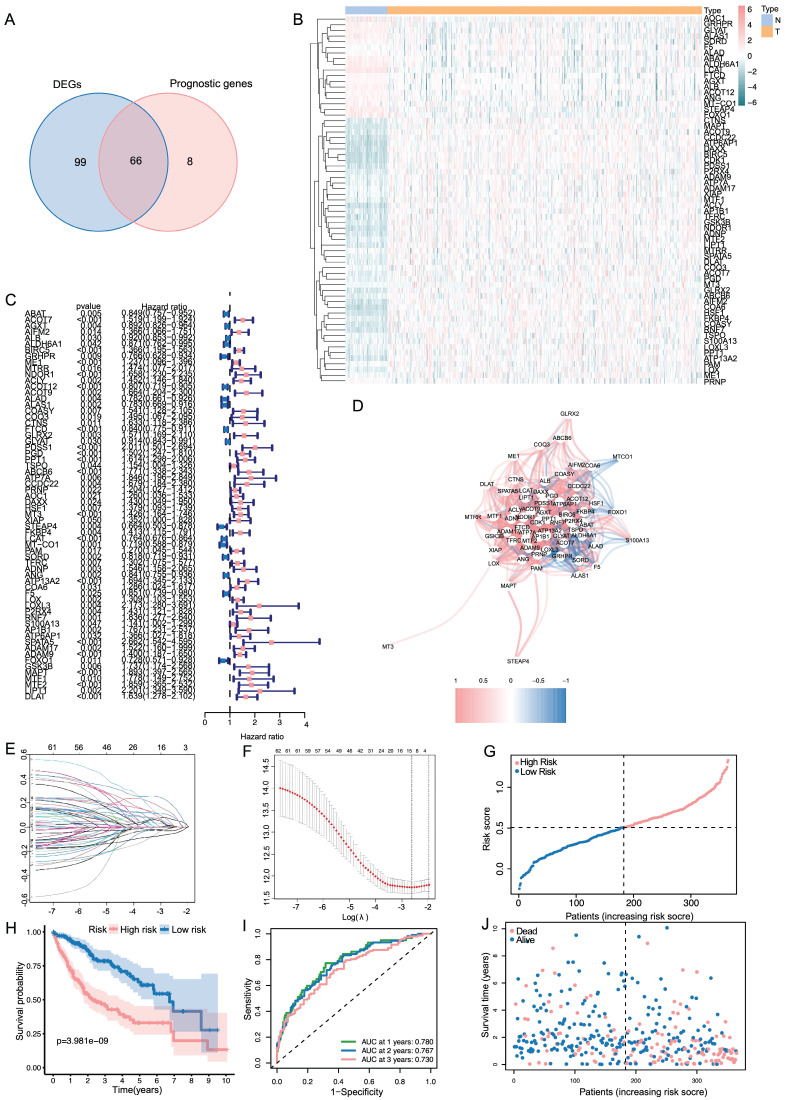
Constructions of CRGs signature in LIHC patients. (A) Venn plot conveys that there are 65 genes in intersection between the results of DEGs and prognostic genes. (B, C) Heat map and the forest plot of the 65 genes. (D) The correlation between the 65 genes. (E, F) The LASSO regression analysis and partial likelihood deviance of the 65 genes. (G, J) The distribution of the risk scores and patient living status. (H) Kaplan-Meier survival estimates of OS according to the signature.

The results showed that a total of 154 genes were differentially expressed. Subsequently, we identified 73 genes related to prognosis using univariate Cox regression analysis and took an intersection for 65 same genes (**Figure 1A**). Based on the TCGA cohort, **Figure 1B** showed the heatmaps of levels of 65 genes comparing LIHC and non-cancerous liver tissues. As shown in** Figure 1C**, a forest plot illustrating the prognostic effects of these 65 DEGs was presented. As shown in **Figure 1D**, there was a correlation between these genes.

### Establishment and validation of copper metabolism-related prognostic genes

LASSO regression analysis was employed on 65 potential prognostic copper metabolism-related genes, and 12 copper metabolism-related genes remained according to the least partial likelihood deviance. (**Figure 1E, F**). Then, using multivariate Cox regression analysis, an optimal predictive signature containing 12 copper metabolism-related genes was identified (**Figure 1C**). A risk score was produced for each patient to examine the prognostic signature's prediction performance. Each patient's risk score was computed as follows: risk score = (0.06703× Exp BIRC5) + (0.01196 × ExpME1) + (-0.00818 × ExpACOT12) + (-0.03017 × ExpFTCD) + (0.18916 × ExpPDSS1) + (0.11806 × ExpABCB6) + (0.03387 × ExpMT3) + (-0.05582 × ExpLCAT) + (0.00089× ExpLOX) + (0.01486 × ExpADAM9) + (0.09132 × ExpMAPT) + (0.01952 × ExpDLAT). Nine genes were regarded as risk factors, while the remaining three genes were identified as protective factors. Based on the median value of the risk score, patients were separated into two groups: high-risk and low-risk. A scatter plot was used to illustrate the distribution of risk score, OS status, and OS time in LIHC patients (**Figure 1G**). The low-risk group had a lower mortality rate than the high-risk group (**Figure 1H**). As indicated by time-dependent ROC curves, the signature had a remarkable predictive performance in LIHC OS in the TCGA cohort (1-year AUC = 0.780, 2-year AUC= 0.767, 3-year AUC= 0.730; **Figure 1I**).

### Validation of the 10-gene signature in the ICGC cohort

**Figure 2:**
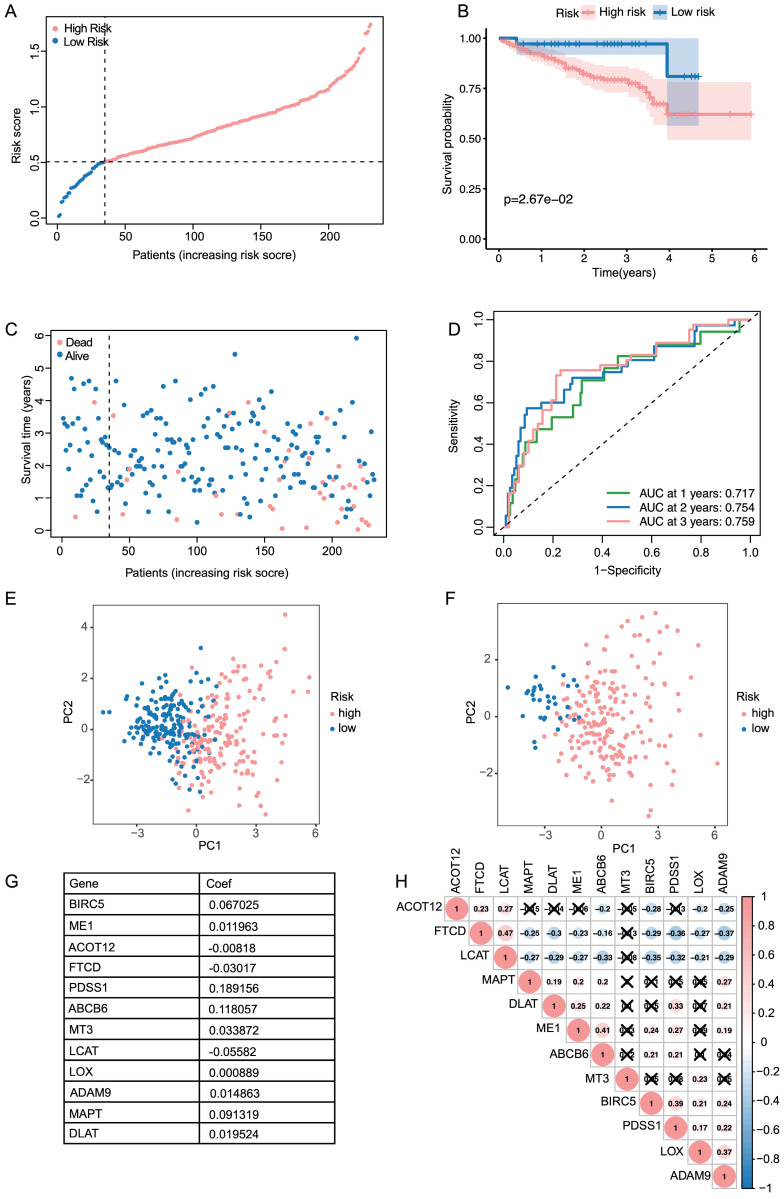
Validation of the signature in the ICGC dataset and evaluation of the 12 genes. (A, C) The distribution of the risk scores and patient survival status. (B) Kaplan-Meier survival estimates the Overall Survival according to the signature. (D) The 1-,2- and 3-year ROC curves of the signature in predicting OS of LIHC patients. (E, F) PCA results of the signature in TCGA and ICGC. (G) The summary sheet of the 12 genes in LASSO. (H)The correlation analysis of the 12 genes in LASSO.

Based on the median value calculated in the same formula as the TCGA cohort, the patients from the ICGC cohort were also grouped into high- or low-risk groups to test the prognostic accuracy of the model constructed based on the TCGA cohort (**Figure 2A**). As with the TCGA cohort, similar Kaplan-Meier survival analysis results were obtained that high-risk group seemed to have a dramatically worse prognosis than the low-risk group (**Figure 2B**). Furthermore, patients in the high-risk group were more likely to die earlier than their counterparts in the low-risk group (**Figure 2C**). According to the time-dependent ROC curves, the AUC reached 0.717 at 1 year, 0.755 at 2 years, and 0.759 at 3 years (**Figure 2D**), indicating good survival prediction performance.

In both TCGA and ICGC cohort, PCA analysis demonstrated that patients were divided into a clear distinction on their risk score (**Figure 2E, F**). Results from the TCGA cohort indicated that patients in the high-risk group presented with more advanced tumor stages, higher tumor grades, increased vascular invasion, and elevated AFP levels (**Table 2**, *p* < 0.05). The specific information and interconnections of the 12 genes included in the calculation formula for risk score based on LASSO regression analysis and multivariate Cox regression analysis were shown in **Figure 2G, H**.

### Functional analysis and immune infiltration analysis of DEGs

**Figure 3:**
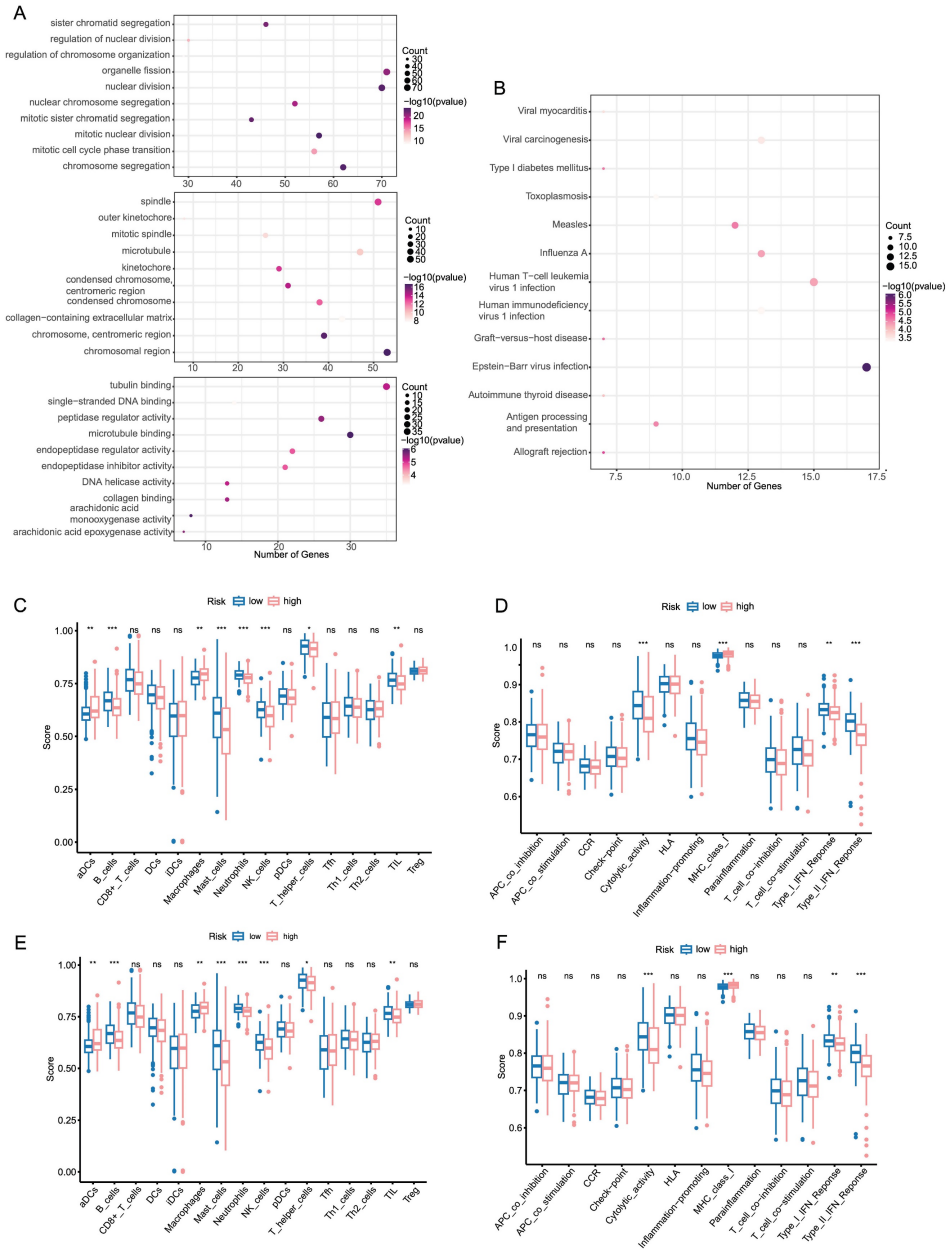
Functional analysis and immune infiltration analysis of DEGs (A, B) Dotplot of GO enrichment and KEGG pathway analysis in DEGs in DEGs. (C, D) Boxplot of immune cells and immune function between high and low risk groups in TCGA. (E, F) Boxplot of immune cells and immune function between high and low risk groups in ICGC.

In order to better understand the functional characteristic and pathways associated with the risk score, GO enrichment and KEGG pathway analysis were performed in DEGs. As in **Figure 3A**, genes were mainly enriched in several cell division-related biological process (BP), such as organelle fission, nuclear division and chromosome segregation. There was a significant enrichment in tubulin binding and microtubule binding among the above-mentioned DEGs regarding molecular function (MF). As for the cellular component (CC) group, the genes were particularly enriched in chromosome region, spindle and microtubule. KEGG pathway analysis also uncovered that the pathways enriched by DEGs were strongly associated with cell cycle, oocyte meiosis and cellular senescence (**Figure 3B**).

To further investigate the relationship between prognostic risk score and immune infiltration status, we quantified the enrichment score of different immune cell subpopulations, related functions or pathways. As illustrated in** Figure 3C, D** the enrichment score of B cells, Mast cells, Neutrophils, NK cells, Cytolytic activity, MHC class 1, type I IFN response and type II IFN response was statistically different between two risk group in TCGA cohort (*P*-value < 0.05). A comparison with the ICGC cohort verified that there was a significant difference of B cells, Neutrophils, NK cells, type I IFN response and type II IFN response between high risk and low risk group (*P*-value < 0.05, **Figure 3E, F**). Notably, similar to the TCGA cohort, in the ICGC cohort score of neutrophils and NK cells showed the most statistically significant differences between two risk groups. In accordance with expectations, high-risk scores were associated with an absence of immune infiltrating cells including B cells, Neutrophils and NK cells, while the score of macrophages was opposite.

### Consensus clustering identified two clusters of patients with LIHC

**Figure 4:**
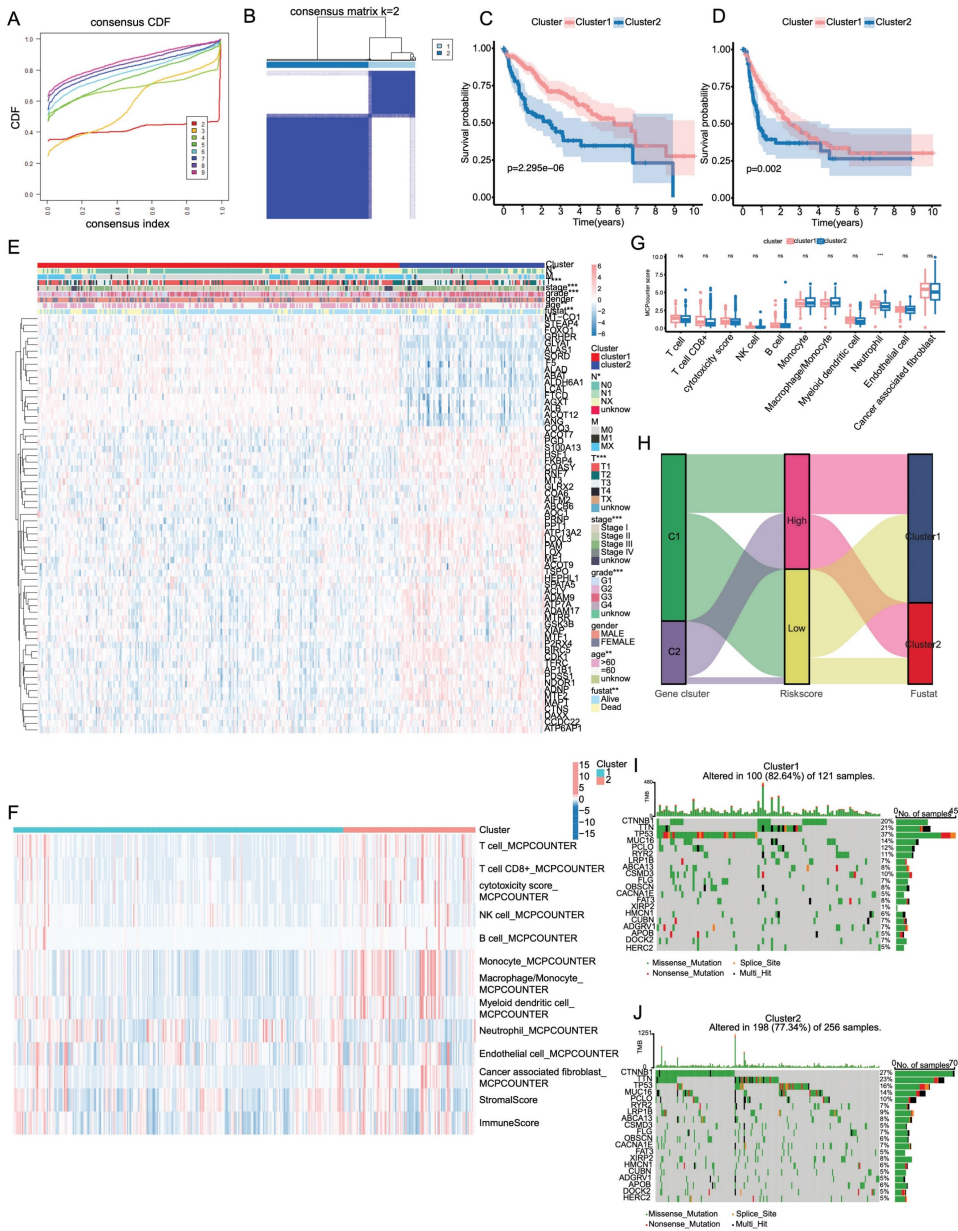
Analysis of the consensus cluster of patients with LIHC. (A, B) Consensus matrix CDF shows that k=2 is the most appropriate selection to divide LIHC patients into different cluster. (C, D) Kaplan-Meier survival estimates of OS and DFs in LIHC according to the two clusters. (E) Heatmap of the clinicopathological characteristics and the expression of CRGs between clustering subgroups. (F) Heatmap of distribution of immune cells and stromal score in two subgroups. (G) Barplot of immune cells between two clusters. (H) Alluvial plot of the gene cluster and lasso subgroup (I, J) The waterfall plot of somatic mutation features established with two clusters. Each column represented an individual patient. The upper barplot showed TMB, the number on the right indicated the mutation frequency in each gene. The right barplot showed the proportion of each variant type.

An analysis of the consensus cluster consisting of 65 genes associated with cuproptosis was conducted and we identified two new clusters of patients with LIHC based on the expression of prognostic genes related to cuproptosis. In **Figure 4A**, we demonstrated the diverse trends in the cumulative distribution function (CDF) of consensus clustering from k = 2 to 9 in the TCGA dataset. Besides, k = 2 appeared to be the most appropriate selection to divide LIHC patients into different cluster due to clustering stability increasing from k = 2 to 9 (**Figure 4B**). Besides, the OS and DFS of LIHC patients in cluster 2 were significantly lower than those in cluster 1 (**Figure 4C, D**). Additionally, there was statistically significant difference in the clinicopathological characteristics and the expression of CRGs between clustering subgroups (**Figure 4E**). The result showed that the different subgroups weren't related to the M stage and gender but T stage, N stage, TNM stage, tumor grade, age, and survival status were significantly different between the two subgroups.

### Tumor mutation burden and immune infiltration in consensus cluster subgroups

Subsequently, we evaluated the distribution of immune cells and stromal score in two subgroups and presented them in a heatmap (**Figure 4F**). Moreover, a bar plot was applied to illustrate the difference in infiltration levels of immune cells in the consensus cluster subgroups, where neutrophil was enriched in cluster 1 (**Figure 4G**). To uncover the changes in risk score, gene clusters, and survival status, alluvial diagram was utilized to display characteristic changes in LIHC patients (**Figure 4H**). A waterfall plot was created to display the differences in the somatic mutations profile landscape between two subgroups (**Figure 4I, K**). The result demonstrated that the mutation frequency of TP53 was significantly higher in cluster 1 than in cluster 2, whereas CTNNB1 was more frequently mutated in cluster 2.

### Building a predictive nomogram based on clinicopathologic features and risk score

**Figure 5:**
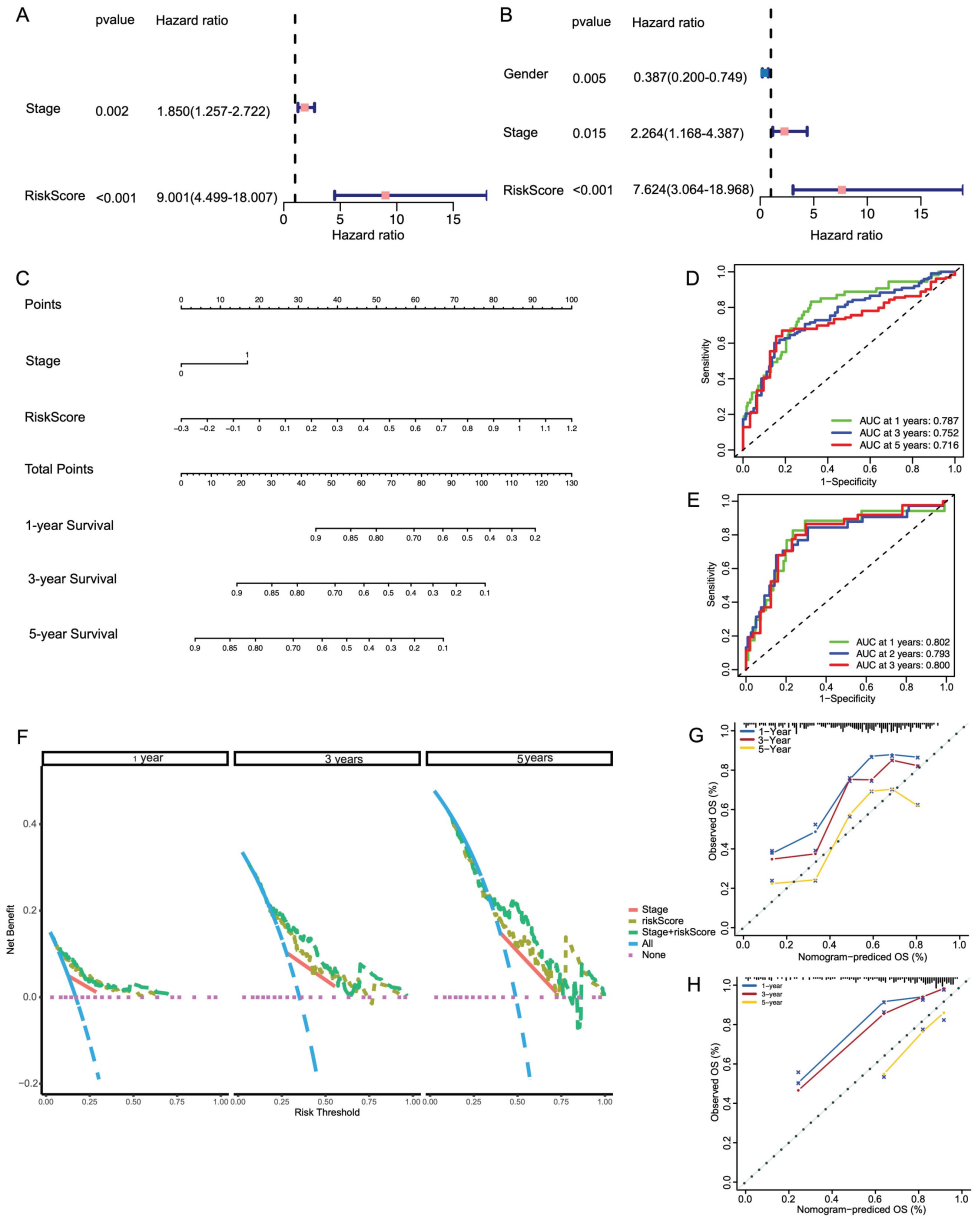
Development of a nomogram for survival prediction of LIHC patients based on signature and clinical characteristics. (A, B) Univariate and multivariate Cox analyses simultaneously demonstrated the independent prognostic value of the risk score. (C) The nomogram combining risk signature and clinicopathological factors. (D, E) AUCs on the nomogram suggested that this model in TCGA and ICGC had higher sensitivity in OS at 1 ,3 and 5 years. (F) Decision curve analysis for stage and risk scores prediction models at 1, 3 and 5 years. (G, H) Calibration plots were established to compare the proposed nomogram with an ideal model in TCGA and ICGC.

Based on the clinicopathologic features and the risk score, a predictive nomogram was constructed to estimate the survival probability. In the TCGA database, univariate and multivariate Cox regression analyses were conducted for the relationship between clinicopathological features and prognosis and whether the risk score was an independent indicator of prognosis. Univariate and multivariate Cox analyses revealed that stage and risk score were independent factors influencing the prognosis of LIHC patients (**Figure 5A, B**). Based on the multivariate Cox regression analysis described above, we constructed a nomogram (**Figure 5C**) for 1-, 3-, and 5-year OS prediction in the LIHC patients, which incorporated two independent factors (stage and risk score). As shown in **Figure 5D, E**, the ROC curve revealed that the combined nomogram could perfectly predict the survival rates for LIHC patients in both TCGA cohort and ICGC cohort. Based on the DCA (**Figure 5F**), we observed that the combined nomogram was most frequent in top position, indicating that it was the most suitable nomogram for predicting the prognosis of individuals with LIHC. In contrast, the combined predictive nomogram could fairly accurately predict the 5-year overall survival rates in both TCGA cohort and ICGC cohort (**Figure 5G, H**).

### CRGs correlate with metastasis and development of human LIHC

**Figure 6:**
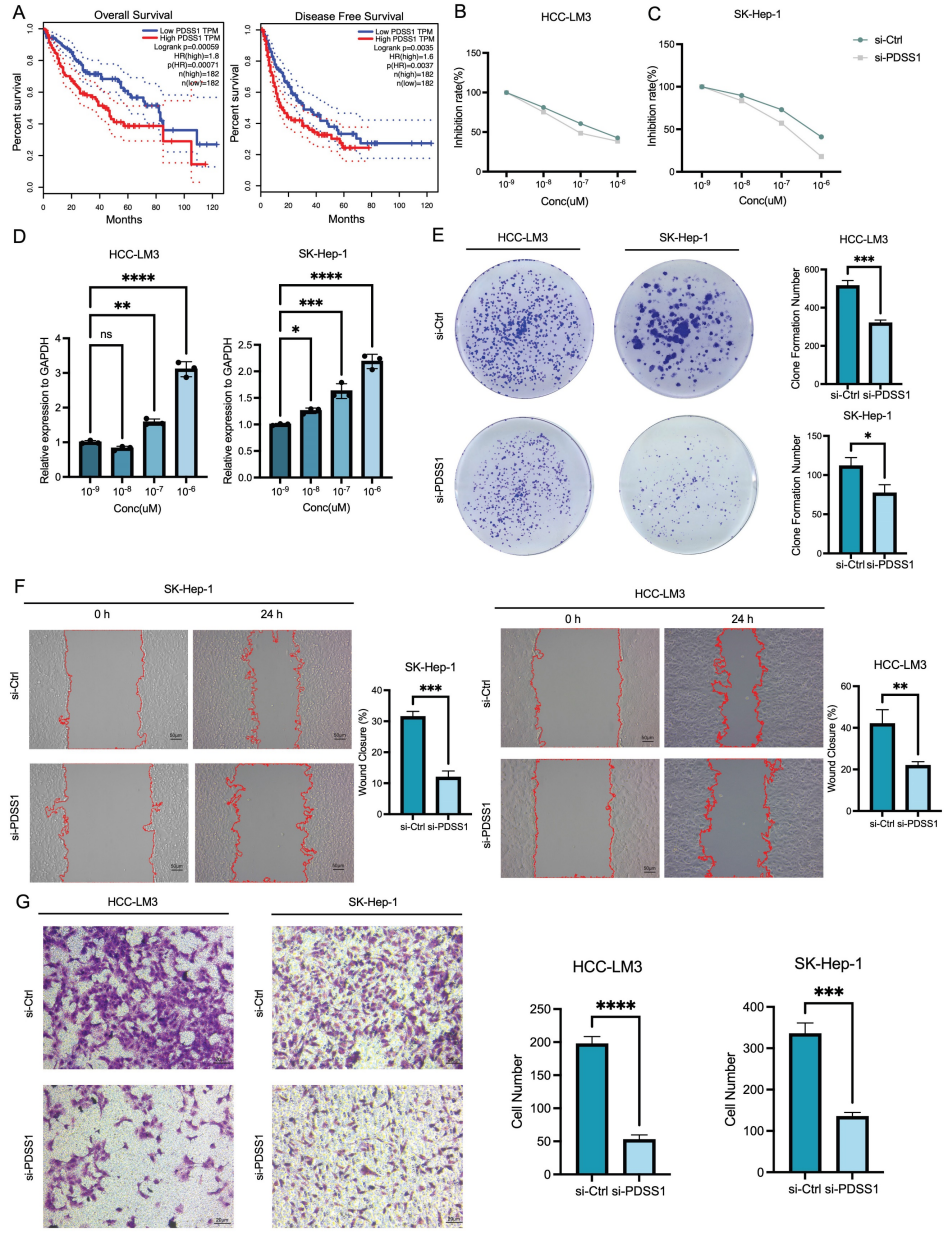
Correlation of CRGs with Metastasis and Development of Human LIHC. (A) OS and DFS curves of PDSS1 expression shows that the high expression of the above-mentioned genes was significantly related to worse prognosis in LIHC patients. (B, C) Two figures are established to show that knocking down PDSS1 in HCC-LM3 and SK-Hep1 cells increased their susceptibility to cuproptosis. (D) The correlation between the expression of PDSS1 and the concentration of CuCl_2_ in HCC-LM3 and SK-Hep1 cells. (E-G) The proliferative and migration capability of HCC-LM3 and SK-Hep1 cells transfected with siRNA were measured using colony formation assay, healing assay and transwell assay.

PDSS1, ABCB6 and MAPT were listed as the top three genes with the highest weights in the predictive nomogram, and we plotted their OS and DFS curves, respectively (**Figure 6A, B, C**). The results also showed that the high expression of the above-mentioned genes was significantly related to worse prognosis in LIHC patients. PDSS1 emerged as the most prominent gene in the LASSO regression model, and we subsequently carried out functional validation specifically targeting it. We investigated the potential of PDSS1 knockdown to enhance the sensitivity of LIHC cell lines to CuCl2. Our results confirmed that knocking out PDSS1 in HCC-LM3 and SK-Hep1 cells increased their susceptibility to cuproptosis (**Figure 6D**). Additionally, we observed an upregulation of PDSS1 expression in HCC-LM3 and SK-Hep1 cells with increasing concentrations of CuCl2, suggesting its involvement in cuproptosis tolerance (**Figure 6E**). To evaluate the impact of PDSS1 knockdown on the proliferation of LIHC cells, we conducted a colony formation experiment, which revealed a significant reduction in the formed colonies upon PDSS1 knockdown in HCC-LM3 and SK-Hep1 cells (**Figure 6F**). Additionally, through Transwell assay, we observed a significant suppression of migration ability in HCC-LM3 and SK-Hep1 cells upon PDSS1 knockdown (**Figure 6G**).

### The landscape of cuproptosis in TME cells in LIHC

**Figure 7:**
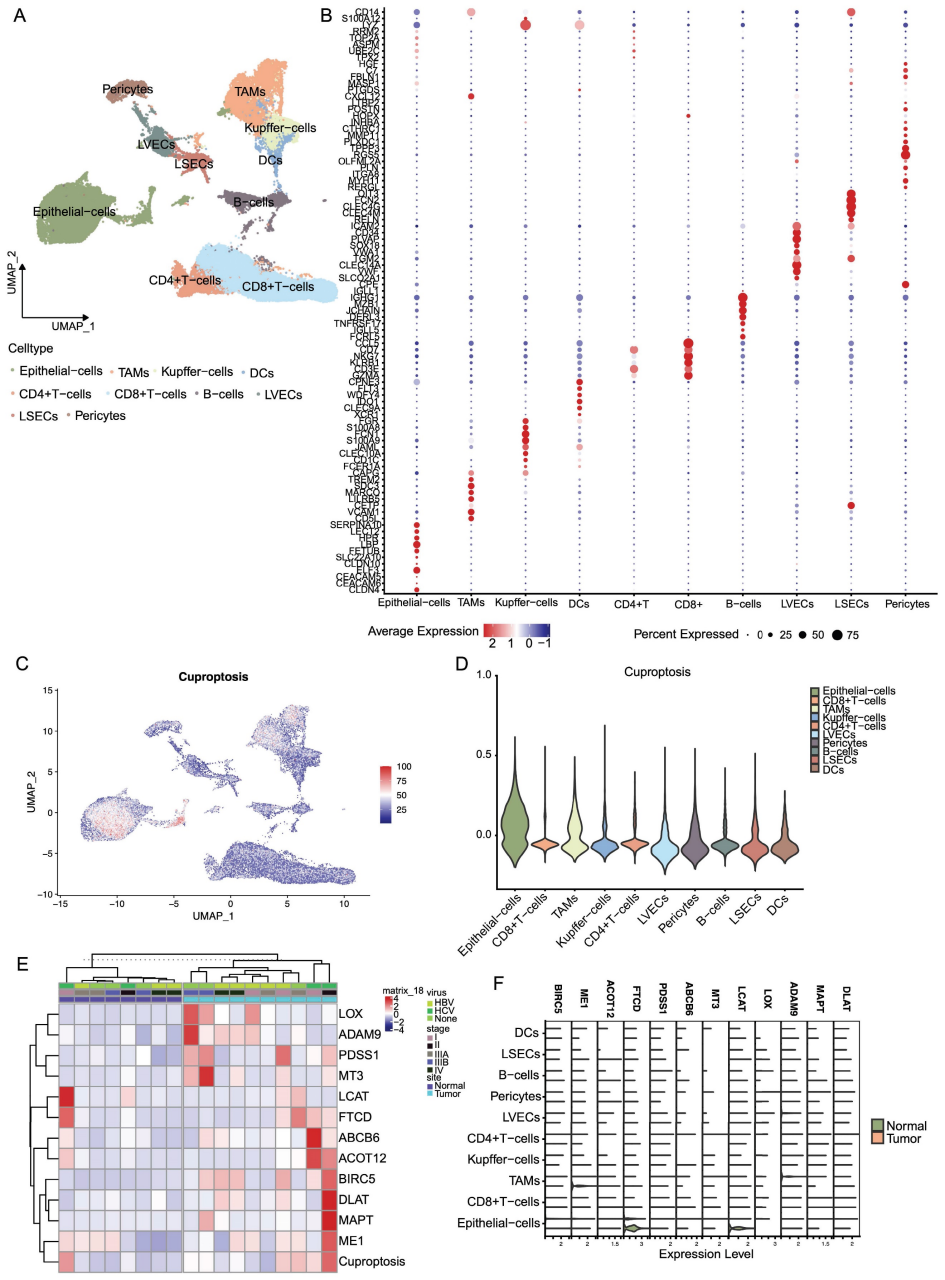
The landscape of cuproptosis in TME cells in LIHC. (A) Clustering analysis on ten distinct cell types. (B) Expression profiles of specific marker genes for each cluster of cells. (C, D) Featureplot and Vlnplot of expression of CRGs in the 10 cells. (E) Heatmap of gene expression in LIHC according to the viral infection status and tumor stage. (F) Expression of CRGs in different cells.

We obtained scRNA-seq data of the tumor microenvironment in LIHC from the GSE149614 dataset and performed clustering analysis on ten distinct cell types, including epithelial cells, CD4+ T cells, CD8+ T cells, B cells, TAMs, Kupffer cells, DCs, LVECs, LSECs, and pericytes (**Figure 7A**). Furthermore, we visualized the expression profiles of specific marker genes for each cluster of cells (**Figure 7B**). To explore the distribution of prognostic genes associated with cuproptosis, we employed the AddModuleScore function in Seurat to score their expression levels. Results from VlnPlot and FeaturePlot confirmed that the CRGs are predominantly expressed at high levels in epithelial cells and TAMs (**Figure 7C, D**). Furthermore, our analysis revealed substantial variations in the average RNA expression of CRGs among LIHC samples across different categories of sample type, viral infection status, and tumor stage. Significantly higher expression levels of particular CRGs associated with poor prognosis were observed in tumor samples compared to normal samples. Additionally, distinct patterns of gene expression were observed in LIHC samples based on their viral infection status and tumor stage, implicating the potential involvement of copper death-related genes in LIHC progression and response to viral infections (**Figure 7E**). On the contrary, as shown in** Figure 7F**, the expression of CRGs which were associated with good prognosis, was relatively high expressed in normal samples.

### Characteristic profiles of cells with different PDSS1 expressions

**Figure 8:**
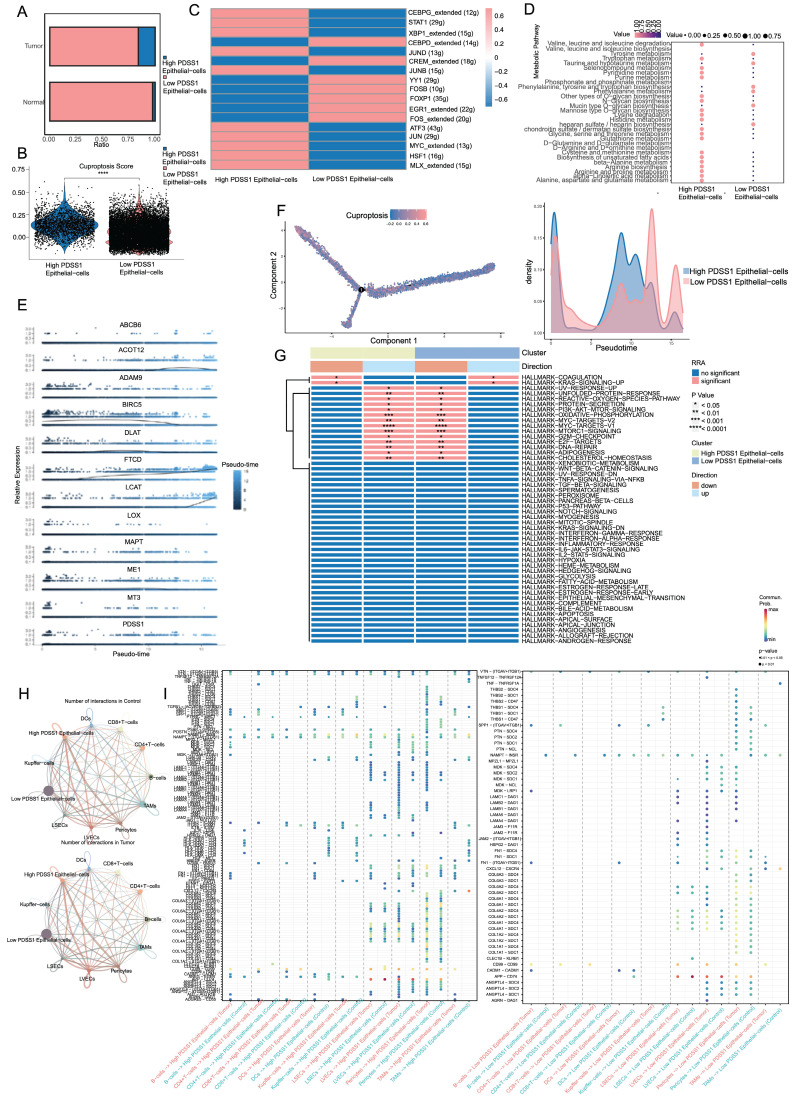
Characteristic Profiles of Cells with Different PDSS1 Expressions. (A) Epithelial cells are categorized into 2 groups according to the expression of PDSS1. (B, C) Expression of cuproptosis gene and TFs in High PDSS1 group and Low PDSS1 group. (D) Character of Metabolites in epithelial cells with different levels of PDSS1 expression. (E, F) Pseudotime and single cell trajectory analysis of genes in CRGs. (G) Heatmap of GSVA analysis (H) Intercellular communication network plot of high PDSS1 expression epithelial cells with surrounding cells. (I) Cell chat mediated by Ligand-receptor.

Based on the expression of PDSS1, we categorized the epithelial cells into two groups: PDSS1 high-expressing epithelial cells and PDSS1 low-expressing epithelial cells (**Figure 8A**). Subsequently, we conducted an exploration of their respective characteristics. As shown in **Figure 8B**, the score of the cuproptosis gene set was also higher in cells with high PDSS1 expression, indicating that the characteristic performance of this group of cells can reflect the impact of cuproptosis on the LIHC cells. Furthermore, we identified the unique TFs regulatory characteristics of the subpopulations through SCENIC and calculated their average regulatory activity. The results confirmed that in epithelial cells with high PDSS1 expression, the regulatory activity of TFs related to tumor progression was significantly enhanced (**Figure 8C**). Given that changes in metabolites were believed to be closely related to cuproptosis, we sought to explore changes in epithelial cells with different levels of PDSS1 expression. Using the scMetabolism package, we found that almost all metabolites are enriched in epithelial cells with high PDSS1 expression, suggesting that PDSS1 constructed a high metabolic tumor microenvironment, thereby affecting other cells (**Figure 8D**). Then, Monocle2 was utilized to perform pseudotime and single cell trajectory analysis. Pseudotime analysis of gene expression in the risk model revealed that the expression levels of FTCD and LCAT, associated with good prognosis, were higher in the late stages of tumor development. This suggested that cuproptosis played different roles at different stages of tumor progression (**Figure 8E**). As shown in** Figure 8F**, epithelial cells with high PDSS1 expression mainly appear in the middle stages of tumor development. Furthermore, through GSVA analysis, we found that the functional enrichment of epithelial cells with high PDSS1 expression was mainly concentrated in the Coagulation and KRAS signaling pathways (**Figure 8G**). The interaction between epithelial cells and immune cells or stromal cells in the tumor microenvironment had a significant impact on tumor development. Through CellChat analysis, we found that epithelial cells with high PDSS1 expression in both tumor tissues and normal tissues had stronger cell communication with surrounding cells (**Figure 8H**). Through ligand-receptor analysis of cell communication, we found that cells with high PDSS1 expression mainly interact with endothelial cells and pericytes through the extracellular matrix and collagen metabolism (**Figure 8I**).

## Discussion

The significance of copper ions in the treatment of hepatocellular carcinoma (HCC) is an area of active research. Copper ions were shown to possess anti-tumor properties, which induced oxidative stress in cancer cells, causing DNA damage and apoptosis (programmed cell death). Recent studies have revealed the dual roles of copper in promoting and suppressing HCC progression 10, 11. Previous study found a significant upregulation in the expression of copper transporter genes in HCC, indicating that restricting copper homeostasis effectively hindered the growth of HCC cell lines 12. Accordingly, targeting copper-dependent vulnerabilities could reveal innovative approaches for HCC treatment. The liver plays a central role in copper metabolism, controlling the biological processes of copper while synthesizing and secreting copper-binding proteins 13. Due to its active involvement in copper metabolism, the liver is particularly susceptible to imbalances in copper levels.

Excess copper can cause liver damage and diseases such as Wilson disease, while copper deficiency is observed in disorders of lipid metabolism, including nonalcoholic liver disease and alcohol-related cirrhosis 6. Given the interplay between liver physiology and copper metabolism, copper homeostasis may have broader implications for the progression of HCC.

In our study, we conducted a comprehensive analysis of 200 genes linked to copper metabolism and cuproptosis in HCC patients, utilizing publicly available datasets. Our findings revealed that 65 CRGs significantly impacted the prognosis of HCC patients. To assess the survival risk of HCC patients, a predictive model is developed with the Lasso method and a nomogram, based on the identification of 12 critical genes using the Lasso method. Additionally, we validated our findings in HCC samples and identified potential targets closely associated with immune cell infiltration, including B cells, neutrophils, NK cells, type I IFN response, and type II IFN response. Taken together, these results underscore the pivotal roles of CRGs in influencing tumor development, while our prediction model offers a valuable tool for clinicians to further prognosticate HCC patients.

The scRNA-seq has emerged as a potent tool in the field of oncology, facilitating the analysis of intricate cell populations and the development of clinical diagnostic markers 6. In the context of HCC, in-depth exploration of the immune cell landscape within both intrahepatic and tumor tissues has provided valuable insights into disease progression. Leveraging a series of single-cell analysis techniques, our findings reveal markedly elevated expression levels of specific CRGs associated with adverse prognoses in tumor samples as compared to their normal counterparts. Furthermore, we have uncovered distinct patterns of gene expression in LIHC samples, stratified by viral infection status and tumor stage. These findings suggest the potential involvement of copper-regulated genes in LIHC progression and their role in responding to viral infections. Consequently, these genes may hold significant promise as valuable biomarkers for predicting the effectiveness of immunotherapeutic interventions in patients battling HCC.

Besides, we have identified PDSS1 as a key regulatory factor inducing cuproptosis in HCC cells, supported by rigorous experimental validation. This discovery marked significant progress in understanding the molecular mechanisms of cuproptosis pathways. Utilizing single-cell RNA sequencing technology, we were able to dissect the complex interactions and mechanisms between different cell subtypes within the HCC tumor microenvironment and their responses to copper stress. This detailed characterization provides a foundational basis for the theoretical support of targeted drug design related to cuproptosis.

In our study, the application of scRNA-seq has profoundly characterized tumor cells with high PDSS1 expression, revealing unique transcriptional and metabolic features, as well as interactions with the immune microenvironment. This in-depth analysis has also uncovered the adaptive mechanisms by which tumor cells withstand copper-induced stress, directing future efforts to address drug resistance associated with cuproptosis.

Furthermore, our research has explored the expression patterns of various cuproptosis-associated genes across different stages of tumor progression, offering a temporal perspective on how these genes facilitate the evolution of the tumor landscape. Understanding these dynamics is crucial for developing targeted therapies that could disrupt these processes and potentially halt tumor progression.

It is essential to acknowledge limitations of this study. Firstly, to elucidate the impact of PDSS1 genes on HCC proliferation and metastasis, *in vivo* experiments are imperative. Furthermore, despite certain validation experiments related to copper metabolism genes in HCC cells, additional investigations are warranted for HCC samples. These investigations will provide valuable insights for scientists delving deeper into the intricate relationship between copper metabolism and HCC tumor progression. Additionally, while our study revealed correlations between CRGs, tumor immune cell infiltration, and immune checkpoints at the single-cell mRNA level, it remains uncertain whether targeting critical CRGs can reverse immune escape and immune therapy resistance. Lastly, for validation and enhancement of our prognostic model, multicenter clinical trials with substantial sample sizes are essential prerequisites.

Our findings not only elucidate prognostic value of PDSS1 in HCC but also deepen our understanding of the tumor microenvironment and the metabolic dependencies of cancer cells. This comprehensive view could lead to innovative strategies targeting the specific vulnerabilities of high PDSS1-expressing cells, such as inhibiting their active interactions with endothelial cells and suppressing their vibrant metabolic states, potentially revolutionizing HCC treatment.

## Supplementary Material

Supplementary table.

## Figures and Tables

**Table 1:** Clinical information of the LIHC patients included in this study.

	TCGA	ICGC(LIRI-JP)
Number of patients	365	231
Age (median, range)	61 (16-90)	69 (31-89)
Gender		
Female	119	61
male	246	170
Grade		
Grade1-2	230	NA
Grade3-4	130	NA
Unknown	5	NA
Stage		
Stage I	170	36
Stage II	84	105
Stage III	83	71
Stage IV	4	19
Unknown	24	0
Survival status		
OS days	598.5 (0-3675)	780 (10-2160)
AFP		
<=200	201	NA
>200	75	NA
Unknown	89	NA
Vascular tumor		
Macro/Micro	106	NA
None	205	NA
Unknown	54	NA

**Table 2:** Clinical information of the patients in high- and low-risk groups in the two cohorts.

		TCGA			ICGC		
		High Risk	Low Risk	*P*	High Risk	Low Risk	*P*
Gender				0.4133			0.605
	Female	63	56		53	8	
	Male	119	127		143	27	
Age				0.4332			0.0866
	<60	86	79		41	3	
	≥60	96	104		155	32	
Grade				< 0.0001			-
	Grade1-2	95	135		-	-	
	Grade3-4	85	45		-	-	
Stage				0.0001			0.1712
	Stage I-II	111	143		116	25	
	Stage III-IV	59	28		80	10	
Vascular_Tumor				0.0033			-
	None	124	126		-	-	
	Macor/Micro	79	41		-	-	
AFP				0.008			-
	≤200 ng/mL	82	119		-	-	
	>200 ng/mL	44	31		-	-	

## Abbreviations

CRGs: cuproptosis-related genes
LIHC: liver hepatocellular carcinoma
TCGA: The Cancer Genome Atlas
ICGC: International Cancer Genome Consortium
scRNA-seq: single-cell RNA sequencing
TAMs: Tumor associated macrophages
NAFLD: non-alcoholic fatty liver disease
FPKM: Fragments per Kilobase Million
CDF: cumulative distribution function
DEGs: differential expression genes
ROC: receiver operating characteristic
qRT-PCR: Quantitative real-time PCR
PCA: Principal Component Analysis
PCs: principal components
UMAP: uniform manifold approximation and projection
GO: gene ontology
KEGG: Kyoto Encyclopedia of Genes and Genomes
CDF: cumulative distribution function
