# Supplementary material for: Screening of PDSS1 as a Potential Biomarker for Hepatocellular Carcinoma Based on a Copper-Related Prognostic Signature through Bulk and Single-cell RNA-sequencing Analysis
Source: J Cancer. 2024 Jul 22;15(15):5028–45. doi: 10.7150/jca.96867 (PMC11310869; doi:10.7150/jca.96867)
Supplement: Supplementary file 1 — Supplementary table. [file jcav15p5028s1.pdf]

Supplementary Table 1: List of Genes Associated with Cuprotpsis in this Research.

| | | | | | |
|---|---|---|---|---|---|
| ABAT | COASY | TSPO | MT1G | ACR | TP53 |
| ACOT7 | COQ2 | UGT1A1 | MT1H | ADNP | TYR |
| AGXT | COQ3 | UROS | MT1HL1 | ANG | AP1B1 |
| AIFM2 | COQ7 | VPS9D1 | MT1M | AOC2 | AP1S1 |
| ALB | COX10 | ABCB6 | MT1X | APOA4 | ATP6AP1 |
| ALDH6A1 | COX15 | ANKRD9 | MT3 | ATP13A2 | ATP6V0A2 |
| AOC3 | CPOX | APP | MT4 | CCS | COG2 |
| BDH2 | CTNS | ARF1 | NFE2L2 | COA6 | SPATA5 |
| BIRC5 | FECH | ATOX1 | SNCA | COX11 | TMPRSS6 |
| GCLC | FTCD | ATP7A | COMMD1 | CUTA | ADAM10 |
| GRHPR | GCLM | ATP7B | CUTC | DBH | ADAM17 |
| HACL1 | GLRX2 | CCDC22 | XIAP | DCT | ADAM9 |
| HPGD | GLYAT | COX19 | STEAP2 | F5 | AKT1 |
| ME1 | GPX1 | MT2A | STEAP3 | F8 | APC |
| ME3 | GSTA1 | PRND | STEAP4 | GPC1 | CASP3 |
| MTRR | ISCU | PRNP | SLC11A2 | HAMP | CCND1 |
| NDOR1 | MLYCD | SCO1 | COX17 | LACC1 | FOXO1 |
| NNT | MOCOS | SCO2 | CP | LOX | FOXO3 |
| NOX4 | MOCS2 | SLC31A1 | FKBP4 | LOXL1 | GSK3B |
| PPOX | MTHFD2 | SLC31A2 | HEPH | LOXL3 | JUN |
| SPR | NFE2L1 | AOC1 | HEPHL1 | LOXL4 | MAPT |
| WWOX | NFS1 | AQP1 | MMGT1 | MOXD1 | MDM2 |
| ACLY | OXSM | AQP2 | PARK7 | MOXD2P | MT1JP |
| ACO2 | PDHB | BACE1 | AANAT | MTCO2P12 | MT1L |
| ACOT1 | PDSS1 | BECN1 | ATP5F1D | OR5AR1 | MTF1 |
| ACOT12 | PDSS2 | CYP1A1 | CDK1 | P2RX4 | MTF2 |
| ACOT2 | PGD | DAXX | IL1A | RNF7 | PIK3CA |
| ACOT4 | PGLS | HSF1 | LCAT | S100A12 | PTEN |
| ACOT9 | PPT1 | MAP1LC3A | LOXL2 | S100A13 | SP1 |
| ALAD | SDHA | MT1A | MT-CO1 | S100A5 | STEAP1 |
| ALAS1 | SDHB | MT1B | PAM | SNAI3 | |
| ALAS2 | SDHC | MT1DP | SOD3 | SNCB | |
| ALDH1L1 | SDHD | MT1E | SORD | SNCG | |
| BLVRA | SOD1 | MT1F | TFRC | SUMF1 | |
